# Characterization of Epoxy Composites Reinforced with Wax Encapsulated Microcrystalline Cellulose

**DOI:** 10.3390/polym8120415

**Published:** 2016-11-30

**Authors:** Yuanfeng Pan, Ying Pan, Qingzheng Cheng, Yi Liu, Charles Essien, Brian Via, Xiaoying Wang, Runcang Sun, Steven Taylor

**Affiliations:** 1 Forest Products Development Center, School of Forestry and Wildlife Sciences, Auburn University, Auburn, AL 36849, USA; jnupyf@hotmail.com (Y.P.); qzc0007@auburn.edu (Q.C.); cze0017@auburn.edu (C.E.); 2 State Key Laboratory of Pulp and Paper Engineering, South China University of Technology, Guangzhou 510640, China; xyw@scut.edu.cn; 3 College of Chemical Science and Engineering, Qingdao University, Qingdao 266071, China; panying@qdu.edu.cn; 4 College of Material Science and Technology, Beijing Forestry University, Beijing 100083, China; liuyi.zhongguo@163.com; 5 Department of Biosystems Engineering, Auburn University, Auburn, AL 36849, USA; taylost@auburn.edu

**Keywords:** paraffin wax, microcapsules, microcrystalline cellulose, surface property, epoxy composites

## Abstract

The effect of paraffin wax encapsulated microcrystalline cellulose (EMC) particles on the mechanical and physical properties of EMC/epoxy composites were investigated. It was demonstrated that the compatibility between cellulose and epoxy resin could be maintained due to partial encapsulation resulting in an improvement in epoxy composite mechanical properties. This work was unique because it was possible to improve the physical and mechanical properties of the EMC/epoxy composites while encapsulating the microcrystalline cellulose (MCC) for a more homogeneous dispersion. The addition of EMC could increase the stiffness of epoxy composites, especially when the composites were wet. The 1% EMC loading with a 1:2 ratio of wax:MCC demonstrated the best reinforcement for both dry and wet properties. The decomposition temperature of epoxy was preserved up to a 5% EMC loading and for different wax:MCC ratios. An increase in wax encapsulated cellulose loading did increase water absorption but overall this absorption was still low (<1%) for all composites.

## 1. Introduction

In the polymer science field, epoxy polymers offer great versatility and an ample range of enhanced properties, such as stiffness, specific strength, dimensional stability, chemical resistance, and strong adhesion within the matrix [1]. However a long-standing challenge of the utilization of the epoxy is how to toughen this brittle polymer without sacrificing other important properties [2]. The advancement in the use of fillers or reinforcements within the epoxy matrix is a common practice. Some of the enhanced properties are brittleness and modulus [1,3,4].

Cellulose comes from renewable resources which are abundant and readily available in nature. Cellulose provides a stable backbone of β (1→4) linked d-glucose units which are capable of forming strong hydrogen bonds leading to the formation of crystal fibers [5]. Other advantages of cellulose particles over traditional materials, such as glass, talc, and mica, include acceptable specific strength properties, low density, enhanced energy recovery, non-toxicity, and low production costs [6,7,8]. The tensile strength of cellulose is particularly important and can improve composite mechanical properties [9,10]. However because of the hydrophilic nature of cellulose particles, there is increased moisture absorption followed by dimensional change [11,12,13,14]. This property results in poor compatibility between cellulose and hydrophobic polymer matrices, such as epoxy.

The efficiency of cellulose reinforced composites depends on the ability to transfer stress from the matrix to the cellulose fiber [13,15]. This stress transfer efficiency plays a critical role in the mechanical properties of the composite [5,12,13]. The hydrophilic nature of cellulose derived from the hydroxyl groups on the surface can form inter-macromolecular hydrogen bonds and external bonds with atmospheric hydroxyl groups [16].

One key hurdle with cellulose-based composites is the strong attraction between fibers resulting in unwanted agglomeration. The interface energy and excess of hydroxyl groups result in an increased agglomeration within the composite causing stress concentrations during fiber loading [16,17]. Currently, there are various suitable treatments, including chemical and physical methods have been tried with technical success, but implementation has been restricted due to high cost barriers [18,19]. A potential solution is the encapsulation of cellulose with wax, which could be cost effective given that wax has already been utilized in many composite processes for moisture resistance. Paraffin wax materials can act as a non-polar shell and have been used extensively in wood products, phase change storage materials, pharmaceuticals, and many polymer-based industries [20]. Wax was used for microcapsules as a curing agent in thermoset resins [21]. Wax is a potential commercial candidate due to its high reproducibility, low production cost, improved stability, environmental friendliness, and low initial capital investment [22,23]. It can also reduce the rate of water flow in capillaries [24] and significantly increase the dimensional stability of wet specimens [25]. In another study, wax additives were successfully incorporated into aqueous wood preservatives to reduce checking and improve the appearance of treated wood exposed to outdoor environments [26]. Finally, MCC has been used as a filler in UV-light curable methacrylic-siloxane resin. MCC addition increased the dynamic moduli and decreased the thermal expansion coefficient. The photocurable microcomposites could be used as innovative protective coatings of damaged wood [27].

Recently, a wax encapsulation technique has been developed specifically for microcrystalline cellulose [28,29]. In this study, simple cellulose mixing techniques with paraffin wax was achieved to partially or completely encapsulate cellulose particles. To investigate the effect of the performance of encapsulated cellulose dispersed in the epoxy polymer, Epon 828 and microcrystalline cellulose (MCC) were chosen as the composite materials, with paraffin wax for encapsulation of MCC. The water absorption, mechanical properties, FTIR spectra, and thermal properties of the encapsulated MCC (EMC) reinforced epoxy composites were analyzed. The surface morphology of EMC/epoxy composites was also observed using a scanning electron microscope. The possible applications of these composites may include food preservation and innovative packaging, aero-automotive, electrical, and electronics fields [30].

## 2. Materials and Methods

### 2.1. Materials

Paraffin wax was purchased from Gulf Oil Corporation, Houston, TX, USA (Gulf Wax, Household, C211P-S-TO). The matrix epoxy polymer (EPON 828) (Catalog No. NC9610653, Manufacturer: E. V. Roberts and Associates Inc. 174-1 gallon) was purchased from Fisher Scientific (Pittsburgh, PA, USA). All materials were stored in sealed containers and placed in a refrigerated room (5 °C). Diethylenetriamine (99%, DETA, CAS No. 111-40-0) curing agent was purchased from VWR Chemical Co. (produced by Alfa Aesar Company, Tewksbury, MA, USA). MCC with an average particle size of 90 μm (CAS: 9004-34-6, *M*_W_: 342.3 g/mol; melting point: 260–270 °C; density: 1.27–1.6 g/cm^3^ (20 °C)), was purchased from VWR Chemical Co. (West Chester, PA, USA). It was stored in sealed a container before use to avoid moisture uptake from the environment.

### 2.2. Coating Cellulose Particles

For the coating process [28,29], EMC particles were obtained by mixing cellulose particles with paraffin wax at 70 °C for 20 min at a constant stirring of 300 rpm. Paraffin wax was first placed in a beaker heated to a temperature of 70 °C and the temperature was maintained until the paraffin wax was completely molten. The subsequent coating was conducted by adding MCC at different concentrations and stirring for 20 min at 500 rpm. The mixture was then cooled to room temperature under constant stirring, and final EMC particles were collected. Ratios of 1:2, 1:3 and 1:4 (wax to MCC, by weight) were utilized to vary the wax coverage on the surfaces of the cellulose particles.

### 2.3. Differential Interference Contrast (DIC) Microscopy

DIC Microscopy (Olympus BX51, Tokyo, Japan) was used to observe the morphology of un-coated MCC and MCC after wax encapsulation. The samples were put on a transparent glass slide and another glass slide was used to separate the sample particles in order to observe individual particles. Then micrographs of each sample were taken using different magnifications.

### 2.4. Preparation of EMC Reinforced Epoxy Composites

**Figure 1:**
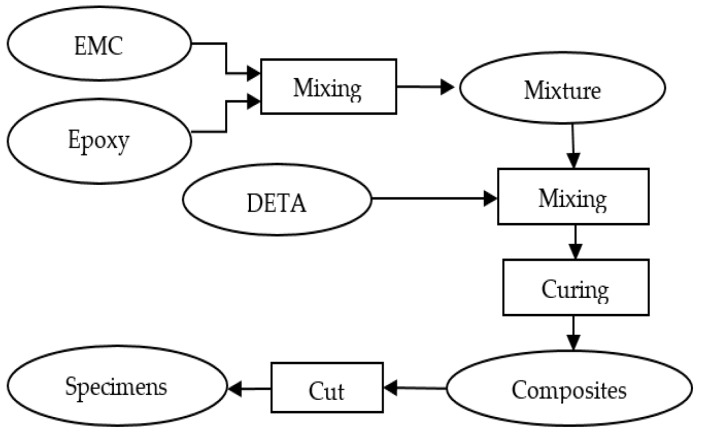
Process flowchart showing the applied fabrication route of EMC/Epoxy composites.

After encapsulation, the EMC particles were added and mixed with epoxy resin prior to cure. EMC loading rates of 0%, 1%, 3%, and 5% (wt % of epoxy resin) were applied at room temperature and mixed with the polymer until fully homogenized. The samples were then obtained by pouring the mixtures into aluminum dishes (10 cm diameter) after DETA was added as a curing agent (10% by weight) to EPON 828 and fully homogenized [31]. The mixtures were then stored at room temperature for 20 min, then 2 h at 50 °C in an oven, and then 1 h at 100 °C. The nominal thickness of the composites was 2 mm. In order to perform mechanical tests and water absorption experiments, the specimens were cut from the cured samples into rectangular bars with dimensions of 50 mm in length and 8 mm in width. The edges of the specimens were smoothed using 60 grit coarse sandpaper. The final specimens were stored in desiccators. The processes of coating, composite, and sample preparations are shown in Figure 1.

### 2.5. Water Absorption

Water absorption of the composite specimens were performed according to ASTM D1037 [32]. First the specimens were dried in an oven at 50 °C for 24 h. Six specimens per treatment were submerged in distilled water at 23 °C for 24 h. Excess water was removed from the specimen surfaces with a dry clean cloth. The specimens were weighed within 1 min after removal from water and then flexure testing was performed (next section). The water absorption (water content, *M*_t_) was determined using the following equation:

*M*_t_ (%) = ((*W*_t_ − *W*_0_)/*W*_0_) × 100

where *W*_t_ is the weight of the sample after soaking and *W*_0_ is the weight of the sample before soaking and after drying in the oven.

### 2.6. Mechanical Properties

Flexure tests performed for both dry and wet samples (before and after 24 h immersion in water absorption test). Flexure tests were conducted using a three point bending protocol at room temperature and at 40% relative humidity. A Z010 Zwick/Roell testing machine (Zwick Roell, Einsingen, Germany) was used with a support distance of 40 mm and testing speed of 1 mm/min (ASTM D790-10) [33]. Six specimens per group were tested.

### 2.7. Scanning Electron Microscopy (SEM)

The cross-section morphology of EMC/epoxy composites after tensile tests were observed using a Zeiss Evo 40XVP scanning electron microscope (SEM, Zeiss, Jena, Germany) to better understand the surface properties of the interaction or bond between Epon 828 and wax or EMC particles. The samples were mounted on aluminum stubs using carbon tape. The samples were then coated with a thin layer of gold to prevent charging before the observation by SEM. The accelerating voltage was 20 kV.

### 2.8. Fourier Transform Infrared Spectroscopy

Fourier transform infrared spectroscopy (FT-IR) measurements were performed using a Perkin–Elmer Spectrum 400 instrument (Waltham, MA, USA) fitted with a single reflectance ATR diamond probe. The samples (MCC, EMC, wax, neat epoxy, neat cured epoxy resin, and EMC/epoxy composite) were measured immediately after manufacture to avoid samples absorbing moisture from the atmosphere.

### 2.9. Thermal Analysis

In order to study the thermal stability and changes in degradation patterns of MCC, neat EPON 828 and their composites, thermogravimetric analyses (TGA) was carried out using a TGA Q5000 instrument (TA Instruments, New Castle, DE, USA) under nitrogen (20 mL/min) from room temperature to 700 °C and at a heating rate of 15 °C/min.

### 2.10. Data Statistical Analysis

The data of water absorption and mechanical tests were analyzed using SAS (version 9.4, SAS Institute Inc., Cary, NC, USA). Analysis of variance (ANOVA, α = 0.05) was used to examine differences of water absorption among the wax to MCC ratios and MCC additions. A paired *t*-test was used to detect the significant differences on the mechanical properties between the composites and the control (pure epoxy).

## 3. Results and Discussion

### 3.1. MCC and EMC Morphology

**Figure 2:**
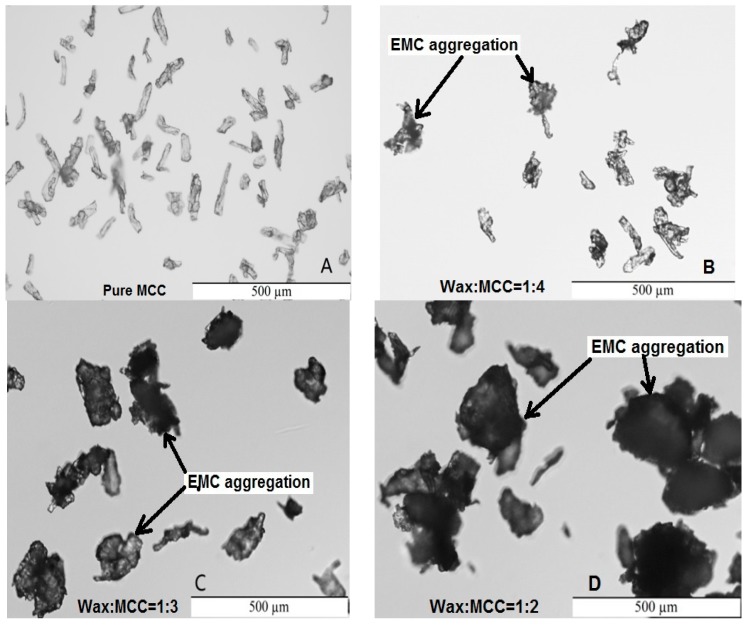
Differential interference contrast (DIC) micrographs of MCC (**A**); and EMC with wax:MCC = 1:4 (**B**); 1:3 (**C**); and 1:2 (**D**), respectively.

Generally, the MCC morphology changed after encapsulation. Figure 2 shows the differential interference contrast (DIC) micrographs of un-coated MCC and MCC after being encapsulated by wax (EMC). Different wax to MCC ratios gave different amounts of wax coverage on the MCC surface. For example, there is a significant amount of wax around the cellulose fiber and more cellulose aggregations when the ratio equals 1:2 (Figure 2D), while less wax coverage results in less cellulose aggregation at a ratio of 1:4 (Figure 2B).

### 3.2. Water Absorption

**Figure 3:**
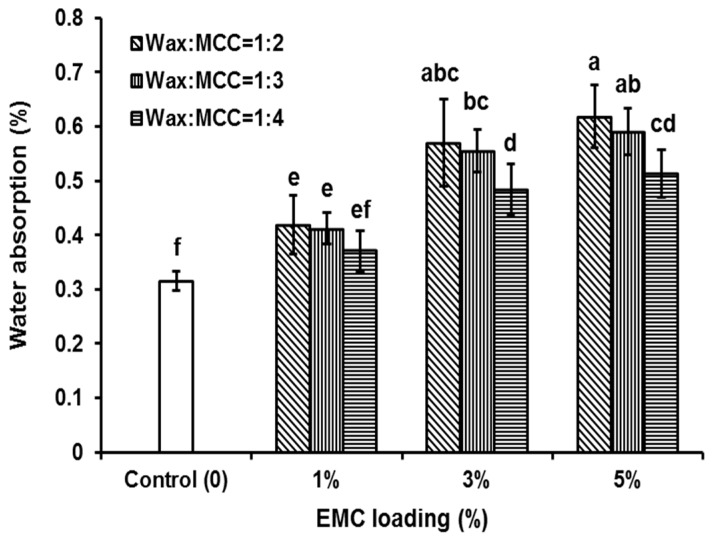
Water absorption (24 h) of epoxy (Control) and EMC/epoxy composites. Means with the same letter (a–f) are not significantly different at 0.05.

Epoxy composites absorbed more water after EMC addition. Figure 3 shows the water content of heat-cured neat epoxy (control) and EMC/epoxy composites (means with the same letter are not significantly different at α = 0.05). It was found that as the EMC loading increased, the water absorption of the composite increased. Generally, after the EMC was added, the water absorption of the composite increased from 0.31% in neat epoxy to 0.62% at 5% EMC loading (ratio of wax to MCC of 1:2).

Water absorption increased as EMC loading increased. Interestingly, for those samples that had the same loading but different ratios of wax to MCC, the water absorption decreased with decreased wax coverage. However, most results were not significantly different, except for the loading of 3% wax and MCC ratios of 1:3 and 1:4 and the loading of 5% wax and MCC ratios of 1:3 and 1:4 (Figure 3). For example, for the 1:2, 1:3 and 1:4 ratios at 5% loading, the water absorption was 0.62, 0.59 and 0.51%, respectively.

Water moisture uptake occurred as cellulose loading increased and the wax to MCC ratio decreased (less wax) from 1:2 to 1:4. It was found that lower wax:MCC ratios (e.g., 1:4 in Figure 1) resulted in less shell coverage. It was hypothesized that more cellulose exposure (no shell coverage) would result in more absorption. However, as wax coverage decreased, the absorption was lower (Figure 3). This suggested that most of the hydroxyl sites on the cellulose surface successfully crosslinked with the epoxy polymer and there were fewer available for water molecules. This also suggested that incomplete wax coverage around the cellulose particle was necessary to allow for crosslinking to the epoxy polymer. For this study, agglomeration of EMC particles was not an issue, supporting that full encapsulation (100% surface coverage) is not needed to prevent agglomeration. This was an important finding since 100% surface coverage would likely inhibit crosslinking between cellulose and epoxy. Former research indicated that neat epoxy can absorb moisture and it can absorb more after hydrophilic cellulose particles were added, although the relative water absorption was low [12,13,14].

### 3.3. Flexure Properties

**Figure 4:**
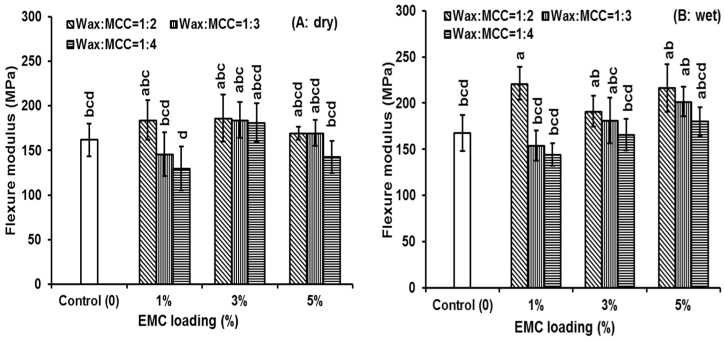
The flexure properties of epoxy and EMC/epoxy composites before (**A**: dry); and after (**B**: wet) immersion in distilled water for 24 h. Means with the same letter (a–d) are not significantly different at 0.05. Both dry and wet samples were compared together.

The wax to MCC ratios, as well as cellulose loadings, could influence the composite mechanical properties. The results are summarized in Table 1 and Figure 4. For the dry samples, the flexural modulus of epoxy did not change much for all wax to MCC ratios and different EMC loadings. For the wet samples, the flexural modulus of epoxy was significantly increased by 1% loading with a 1:2 ratio of wax to MCC and by 5% loading with 1:2 and 1:3 ratios of wax and MCC. These indicated that at a 1% EMC loading and a 1:2 ratio of wax to MCC were good for both dry and wet property reinforcement. This suggests that at a 1% EMC loading and at a 1:2 ratio of wax to MCC, there could be an optimum crosslink density between the cellulose and epoxy matrix. There were decreasing trends of moduli with more EMC additions in the composites (from 1% to 3% and 5% loadings, Table 1 and Figure 4). Similar results were obtained that cellulose might increase the polymer’s moduli and the increment trends were decreased or the moduli might decrease when more cellulose were added [9,10,34]. However, the MCC addition could increase the dynamic moduli of UV-light cured photoresin matrix proportional to the MCC content, which is probably because of the different composite processes and different resins [27].

Lower wax to MCC ratios (less wax) might decrease the epoxy modulus (Table 1), which has the similar changing trends as the water absorption decreases from 1:2 to 1:3 and to 1:4 ratios of wax to MCC for all three EMC loadings (1%, 3% and 5%) (Figure 3). For example, the moduli of the composites with 1% EMC loading and a wax to MCC ratio of 1:4 were significantly lower compared to those of the composites with 1% EMC loading and the wax to MCC ratio of 1:2 for both dry and wet samples (Table 1, 210 vs. 143 for dry, and 221 vs. 144 for wet, respectively). The 1:2 ratio of wax to MCC had less wax coverage and cellulose particle agglomeration than those of 1:3 and 1:4 ratios (Figure 2), but it still had better reinforcement compared to those of 1:3 and 1:4 ratios, which indicated that the wax on the cellulose surfaces could melt and allow more cellulose surfaces to be exposed for bonding to the epoxy matrix. These results support that wax encapsulation was successful at improving cellulose dispersion while still allowing for a bridge between cellulose and epoxy for bonding and reinforcement. It is notable that the addition of EMC could significantly increase the stiffness of wet epoxy composites (Table 1) probably due to the improvement of dimensional stability of wet specimens [22,23,24,25].

### 3.4. SEM Observation of EMC/Epoxy Resin Composites

**Figure 5:**
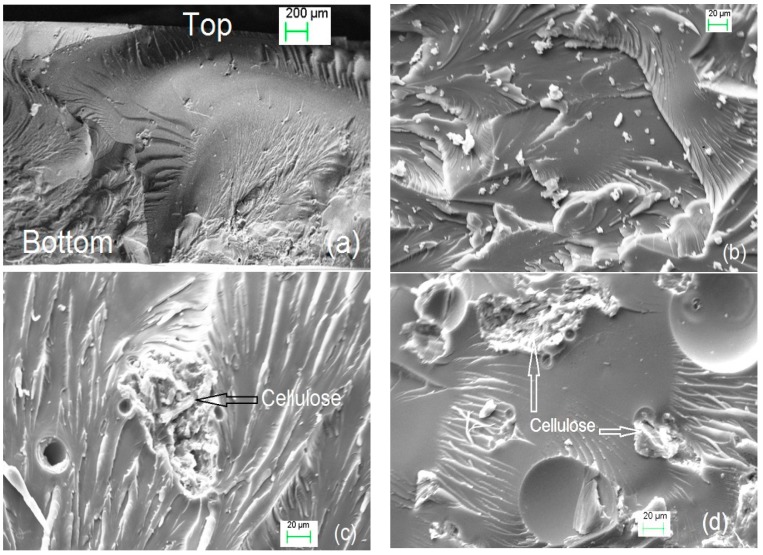
SEM images of the fracture surfaces of (**a**) 1% EMC loading composite with whole thickness; (**b**) neat epoxy; (**c**) 3% EMC loading composite; and (**d**) 5% EMC loading composite.

Scanning electron microscope (SEM) images were used to observe the fractured surface morphology of the EMC/epoxy composites (Figure 5). It was observed that the failure surface of the composites included compression failure on the top and tension failure on the bottom (Figure 4a). The fracture surface of neat epoxy (Figure 4b) was relatively smooth, indicating a brittle failure mode without any ductility, while the fracture surface of the EMC composites (Figure 4c,d) were much rougher than that of the neat epoxy. Meanwhile, some oriented fibril-like materials were clearly observed, indicating some plastic deformation occurred. Compared to neat epoxy, more energy might be needed for crack propagation and for the formation of new surfaces because the plastic deformation and crazing processes could significantly absorb the energy. Similar results were also observed in the other studies [35,36]. The encapsulation with wax provided a thin shell and did not influence the size of the particles in the width or length direction after mixture in the polymer matrix, which also indicated that wax on the cellulose surfaces could melt during the composite processing. The rod-like shapes of MCC were maintained which is important in optimizing composite performance [34]. Some holes or voids were observed in the SEM images (Figure 4c,d), revealing that there were separations between cellulosic rods and the epoxy matrix and that the cellulose as dispersed well in the cross-sections of the composites, supporting the prevention of agglomeration during composite preparation. Other studies have found agglomeration of cellulose to be problematic for a suite of composites. For example, the agglomerations would weaken the mechanical properties and degrade optical performance. In cellulose (10%)/HDPE composites, the agglomerations were responsible for poor adhesion between the fiber and matrix [37].

### 3.5. FT-IR Spectroscopy

**Figure 6:**
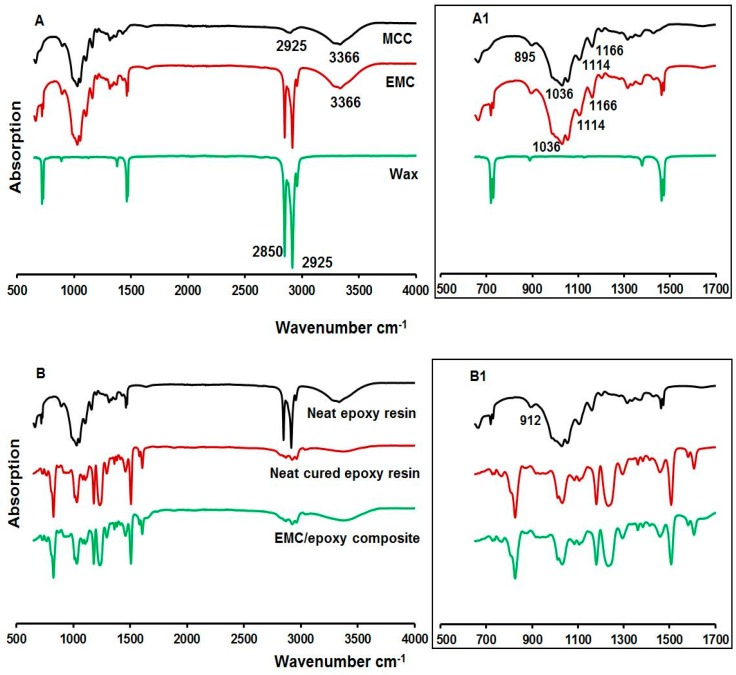
FTIR spectra of MCC, EMC and wax (**A** and zoomed **A1**); and neat epoxy resin, neat cured epoxy resin and EMC reinforced (3% EMC loading) epoxy resin (**B** and zoomed **B1**).

To assess the degree of cure in the matrix and understand the chemical interactions between exposed cellulose and epoxy, characteristic bands were assigned based on a priori data from the literature [38,39]. The spectra of MCC, EMC, and wax are shown in Figure 6A,A1, and the Figure 6B,B1 shows the spectra of the neat epoxy resin, neat cured epoxy resin and EMC reinforced (3% loading) epoxy resin. The band at 895 cm^−1^ for pure MCC is typical of β-glucosidic linkage. The absorption at 912 cm^−1^ was attributable to the unreacted epoxide group characteristic of epoxy resin. Its disappearance in the neat cured epoxy and all composites indicated that all epoxide groups reacted after cure. The strong absorptions between 1000 and 1250 cm^−1^ were assigned to C–O–C (ether) or C–O (H) of cellulose. The absorption at 2925 cm^−1^ was attributable to saturated hydrocarbon groups (C–H). Furthermore, the absorption around at 2850 and 2925 cm^−1^ were attributable to wax [40,41]. Additionally, the bands observed at 3000–3500 cm^−1^ was due to hydroxyl groups.

In EMC-epoxy composites, the epoxide group reacted with –NH2 groups from the amine or hydroxyl groups on the MCC surface [42,43]. It is anticipated that, in this study, there were fewer hydroxyl groups available due to partial encapsulation of MCC. If an epoxide group reacts with a MCC hydroxyl group, the result will be an ether group and a hydroxyl group. The spectra showed that pure MCC, all the neat epoxy resin, and the composites contain hydroxyl groups and ether groups, and their absorptions overlap in the range of 1000–1250 cm^−1^.

### 3.6. Thermal Analysis

**Figure 7:**
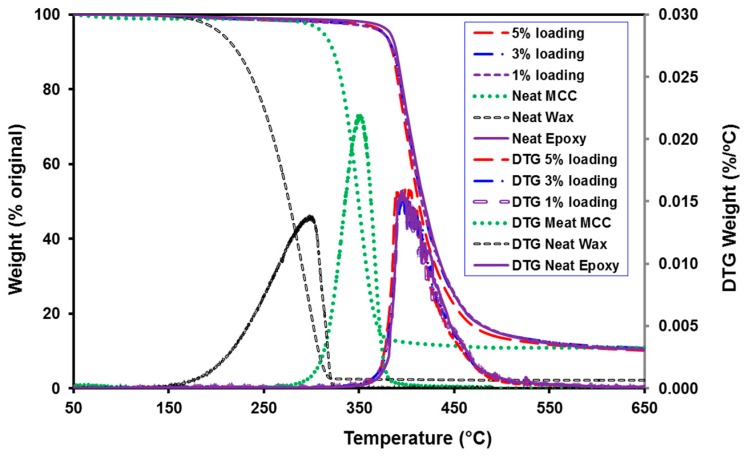
Thermogravimetric analysis (TGA) and their derivative thermogravimetric analysis (DTG) curves of neat MCC, neat wax, neat epoxy and EMC/Epoxy composites.

The thermal stability and changes in degradation patterns of the raw material and the composites were evaluated by thermogravimetric analysis (TGA). Figure 7 shows the thermal decomposition of EMC reinforced epoxy composites (1%, 3%, and 5% loading), cured neat epoxy, neat MCC, and neat wax, as well as their derivative thermogravimetric analysis (DTG) curves. As expected, the wax exhibited the lowest temperature to degradation. While not important for this study, the decomposition of wax at lower temperatures than MCC could be helpful for other composites because the wax could melt and decompose first, resulting in more cellulose exposure to the resin or polymer matrix. When the neat epoxy was analyzed, a superior resistance to higher temperatures was witnessed. The addition of up to 5% EMC did not appear to compromise the thermal degradation, suggesting that epoxy reinforced with EMC could still be used in high-temperature applications. The preservation of thermal resistance with EMC loading may be explained by the insulation properties of epoxy around the EMC particles during temperature loading. Some research also showed that adding cellulose could increase the degradation temperatures of some polymers [34].

## 4. Conclusions

Wax encapsulated microcrystalline cellulose (EMC) allowed for the homogenous dispersion of microcrystalline cellulose (MCC) in the epoxy matrix without much agglomeration. The wax coverage was not a complete sheath, resulting in adequate cellulose exposure for crosslinking between cellulose and epoxy matrix. In summary, the present wax encapsulation method is simple, cost effective, and may not require additional capital during scale up to a manufacturing process. The EMC particles exhibited many advantages such as limited agglomeration, improved hydrophobicity, and homogenous dispersion in non-polar matrices. The EMC could be employed in the epoxy matrix and provide successful reinforcement at only 1% loading. The results indicated that 1% of EMC loading with a 1:2 ratio of wax to MCC was good for both dry and wet property reinforcement, which suggested that there could be an optimum crosslink density between the cellulose and epoxy matrix. The thermal resistance of the epoxy polymer was also not lowered after addition of EMC. The epoxy cellulosic composites utilizing biomass materials could be beneficial for the polymer and wood-related industries by incorporating wasted materials into new environmental friendly products. Furthermore, the cellulosic epoxy composites could enlarge the applications of epoxy polymers, such as in packaging, automotive, and electrical fields.

## Figures and Tables

**Table 1:** The flexure moduli of epoxy and EMC/epoxy composites before (dry) and after (wet) immersion in distilled water for 24 h.

Loading	Wax:MCC	Dry		Wet	
		MOE (MPa) ^1^	Significance ^2^	MOE (MPa) ^1^	Significance ^2^
Control (0)	0:0	159.3 (39.1)		161.7 (29.8)	
1%	1:2	210.1 (35.9)	*	221.3 (39.9)	*
	1:3	165.3 (33.2)		153.9 (36.6)	
	1:4	142.7 (31.2)		144.4 (15.8)	
3%	1:2	191.7 (46.9)		191.4 (37.4)	
	1:3	187.5 (39.5)		185.0 (45.0)	
	1:4	184.0 (44.6)		165.8 (38.0)	
5%	1:2	169.4 (16.2)		216.5 (28.5)	*
	1:3	169.6 (32.7)		201.6 (20.5)	*
	1:4	146.8 (34.4)		180.3 (25.3)	

^1^ The numbers in the parentheses are standard deviations; ^2^ the significant differences are between the composites and the control (pure epoxy) using α = 0.05; * means significant.

## Abbreviations

DIC	Differential Interference Contrast Microscopy
EMC	Encapsulated Microcrystalline Cellulose
FTIR	Fourier Transform Infrared spectroscopy
MCC	Microcrystalline Cellulose
TGA	Thermogravimetric Analyses
